# Stress as a Risk Factor for Substance Use Disorders: A Mini-Review of Molecular Mediators

**DOI:** 10.3389/fnbeh.2018.00309

**Published:** 2018-12-21

**Authors:** Deepika Mukhara, Matthew L. Banks, Gretchen N. Neigh

**Affiliations:** ^1^Department of Anatomy & Neurobiology, Virginia Commonwealth University, Richmond, VA, United States; ^2^Department of Pharmacology & Toxicology, Virginia Commonwealth University, Richmond, VA, United States

**Keywords:** stress, addiction, cocaine, VTA, NAc, drugs

## Abstract

The extant literature supports the role of stress in enhancing the susceptibility of drug abuse progressing to a substance use disorder diagnosis. However, the molecular mediators by which stress enhances the progression from cocaine abuse to cocaine use disorder *via* the mesolimbic pathway remain elusive. In this mini-review article, we highlight three mechanisms by which glucocorticoids (GCs) and the dopaminergic system interact. First, GCs upregulate tyrosine hydroxylase (TH), the rate-limiting enzyme in dopamine (DA) synthesis. Second, GCs downregulate monoamine-oxidase (MAO), an enzyme responsible for DA removal. Lastly, GCs are hypothesized to decrease DA reuptake, subsequently increasing synaptic DA. Based on these interactions, we review preclinical literature highlighting how stress modulates the mesolimbic pathway, including the ventral tegmental area (VTA) and nucleus accumbens (NAcs), to alter cocaine abuse-related effects. Taken together, stress enhances cocaine’s abuse-related effects at multiple points along the VTA mesolimbic projection, and uniquely in the NAcs through a positive feedback type mechanism. Furthermore, we highlight future directions to elucidate the interaction between the prefrontal cortex (PFC) and key intermediaries including ΔFosB, cAMP response element binding protein (CREB) and cyclin-dependent kinase 5 (CDK5) to highlight possible mechanisms that underlie stress-induced acceleration of the progression to a cocaine use disorder diagnosis.

## Introduction

The Diagnostic and Statistical Manual of Mental Disorders (DSM)-V criteria for substance use disorders is defined as “recurrent use of alcohol and/or other drugs causes clinically and functionally significant impairment, such as health problems, disability and failure to meet major responsibilities at work, school, or home” (American Psychiatric Association, 2013). Substance use disorders may range from mild-to-severe and include a variety of substances such as opiates, nicotine, alcohol, cocaine and others, each of which has different mechanisms of action and protein targets. While cocaine exposure does not always progress to a cocaine use disorder diagnosis, a subset of individuals will progress to severe cocaine use disorder or what is referred to as cocaine “addiction” in the preclinical literature. Although epidemiological reports vary, cocaine use disorder is estimated to have an incidence of 0.1% worldwide (Shield et al., 2018). Although the factors that drive progression to substance use disorders are not fully defined, several lines of evidence suggest stress exacerbates susceptibility to the abuse-related effects of drugs (Piazza and Le Moal, 1998; Sinha, 2001; Cleck and Blendy, 2008). For example, neonatal stress selectively enhances the acquisition of cocaine self-administration in rats, but does not augment self-administration when the reinforcer is food (Kosten et al., 2000). Social housing stress in nonhuman primates enhances the reinforcing effects of cocaine in subordinate monkeys (Morgan et al., 2002); however, early life stress produced by maternal separation does not enhance the abuse-related effects of cocaine in nonhuman primates (Ewing Corcoran and Howell, 2010).

Moreover, cumulative adversity is significantly predictive of drug abuse in a dose-dependent manner (Sinha, 2008). In fact, the limbic-hypothalamic-pituitary-adrenal axis (LHPA) axis, responsible for governing the stress response, has substantial overlap with the mesolimbic “reward” pathway involved in reward circuitry (Koob, 2009). The mesolimbic pathway involves dopaminergic projections from the ventral tegmental area (VTA) to the nucleus accumbens (NAcs) and olfactory tubercle in the brain (Quintero, 2013). This pathway is hypothesized to have a critical role in the perception of pleasure and is conceptualized by Koob (2011) to have several key functions: associating meaning to reward-related cues, motivating goal-oriented behavior and general activation. In this mini-review article, we will focus on the impact of stress on cocaine abuse-related effects mediated through the mesolimbic dopamine (DA) “reward” pathway. Given the considerable evidence supporting an impact of stress on substance use disorder susceptibility and relapse, improved understanding of the mechanisms by which stress alters the abuse-related effects of drugs may provide insight into novel molecular targets for therapeutic interventions.

### Underlying Mechanisms of Cocaine Abuse

Cocaine nonselectively binds to all three monoamine transporters (DA, norepinephrine, and serotonin) and prevents the reuptake of these monoamines into the presynaptic terminal thereby enhancing monoamine neurotransmission. Cocaine inhibition of the DA transporter is thought to be the primary mediator of the abuse-related effects of cocaine (Ritz et al., 1987; Volkow et al., 1997). Despite the DA transporter being the primary target for cocaine’s abuse-related effects, repeated cocaine exposure does not alter presynaptic DA transporter availability in either humans (Wang et al., 1997) or nonhuman primates (Czoty et al., 2007). However, repeated cocaine exposure has been shown to increase serotonin and norepinephrine transporter densities in nonhuman primates (Macey et al., 2003; Beveridge et al., 2005; Banks et al., 2008). Furthermore, repeated cocaine exposure downregulates both presynaptic and postsynaptic DA receptors in humans (Volkow et al., 1990, 1993), nonhuman primates (Nader et al., 2006) and rats (Laurier et al., 1994). These cocaine-induced decreases in DA receptors on both pre- and post-synaptic terminals, and the resulting reduced dopaminergic tone, are thought to contribute to the depressive-like symptoms of cocaine withdrawal and relapse of cocaine abuse (Volkow et al., 1993; Thomas et al., 2001).

In substance use disorders, relapse can be triggered by drug-related cues that function as discriminative stimuli to signal contingencies of drug availability and promote drug-taking behavior. For example, following drug-associated cue presentation, the amygdala signals to dopaminergic cell bodies in the VTA (Nestler and Carlezon, 2006; Cleck and Blendy, 2008). These VTA dopaminergic neurons then signal to the NAcs to release DA, which triggers increased gamma-aminobutyric acid (GABA)-ergic input to the thalamus (Koob, 1992; Nestler and Carlezon, 2006). This GABAergic thalamic input leads to hypoactivation of the prefrontal cortex (PFC), impairing judgment and reasoning (Volkow and Morales, 2015). Thus, a combination of increased DA output in the mesolimbic pathway and decreased PFC activation in cortical pathways appear to result in increased drug-taking behavior. Curiously, various types of stressors have been shown to promote drug-taking behavior in preclinical models of drug relapse (Mantsch et al., 2016; Dong et al., 2017), further highlighting the interconnection between stress and reward pathways in the brain.

### Mechanisms of Stress Response

The LHPA influences a variety of functions including the digestive system, immune system, reproductive system, mood and energy expenditure (Vázquez, 1998). The LHPA undergoes self-regulation through feedback and modulates the extrahypothalamic stress neurocircuit (Koob and Kreek, 2007). In addition, the LHPA activates the brain reward circuit (Koob and Kreek, 2007), bridging the interdependent relationship of glucocorticoids (GCs) and the dopaminergic system.

The LHPA is activated following hypothalamic release of corticotropin-releasing hormone (CRH) and vasopressin through a hypophyseal portal system to the anterior pituitary (Aguilera, 2011). CRH may be triggered by either internal or external cues. Synergistically interacting with vasopressin, CRH induces adrenocorticotrophic hormone (ACTH) release by the anterior pituitary. ACTH then acts on the adrenal gland inducing GC secretion into the bloodstream. Cortisol, the primary GC in humans, binds to the GC receptor (GR) in the brain and other end organ tissues facilitating the stress response. The LHPA modulates the stress response through negative feedback on the axis, specifically through negative feedback on the anterior pituitary and hypothalamus that inhibits ACTH and CRH release, ultimately decreasing blood cortisol levels through reduced release.

The GR is a transcription factor, and following translocation to the nucleus, the GR can modulate 10%–20% of genes in the human genome (Oakley and Cidlowski, 2013). While unbound GR remains in the cytosol, in the presence of cortisol, bound GR translocates to the nucleus and interacts with GC response elements (GREs) to modulate transcription (Chrousos et al., 2009). Moreover, GR interacts with other transcription factors, including nuclear factor-κB (NF-κB; Russo et al., 2007) and activator protein-1 (AP-1), which have been implicated in the progression to severe substance use disorder (Hope, 1998; Chrousos et al., 2009).

### Interactions Between Glucocorticoids and the Dopaminergic System

**Figure 1:**
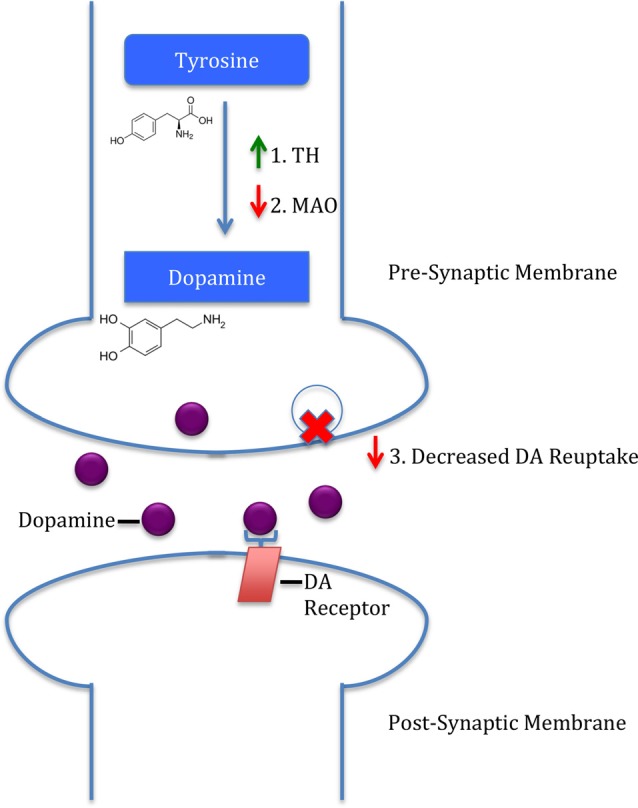
Three mechanisms by which glucocorticoids (GCs) induce dopamine (DA) release. First, GCs upregulate tyrosine hydroxylase (TH), the rate-limiting enzyme in DA synthesis. Second, GCs downregulate monoamine-oxidase (MAO), an enzyme responsible for DA removal. Lastly, GCs are hypothesized to decrease DA reuptake, subsequently increasing synaptic DA.

The interactions between LHPA-induced GC release and the dopaminergic system are pivotal to understanding interactions between stress and substance use disorders. Both stressors and drugs of abuse have been shown to activate the mesolimbic “reward” pathway. For example, both increase glutamate receptor activation of VTA dopaminergic neurons (Cleck and Blendy, 2008). In addition, the LHPA axis also enhances glutamatergic plasticity in the VTA (Stelly et al., 2016). Furthermore, Barrot et al. (2000) have shown that adrenalectomy leading to decreased GC levels resulted in decreased basal and cocaine-induced increase in NAcs shell DA levels. Figure 1 shows three potential mechanisms by which GCs are hypothesized to alter dopaminergic activity. First, GCs increase DA biosynthesis by enhancing tyrosine hydroxylase (TH) activity, the rate-limiting enzyme in DA synthesis (Daubner et al., 2011). This is illustrated by the observation that rats exposed to social isolation have increased TH levels in the NAcs shell (Trainor, 2011). A second mechanism by which GCs are hypothesized to alter dopaminergic activity is through GC-induced reductions in monoamine-oxidase (MAO) activity (Poletto et al., 2011). MAO is another method, in addition to monoamine reuptake by presynaptic transporters as described above, for terminating monoamine neurotransmission. Decreased MAO activity would increase synaptic DA levels and enhance dopaminergic neurotransmission. Lastly, GCs acting at GRs have been shown to regulate DA transporter expression under both basal and cocaine-stimulated conditions (Wheeler et al., 2017). These results are also consistent with reduced DA transporters in rats that underwent early life stress (Meaney et al., 2002). Overall, this literature supports a role of GC regulation of the mesolimbic DA pathway at multiple levels to alter both basal and cocaine-induced dopaminergic neurotransmission.

## VTA

### Increased Glutamatergic Plasticity

Both stress and drugs of abuse have been shown to increase glutamatergic plasticity in the VTA (Saal et al., 2003). Furthermore, exposure to stressful events enhances VTA glutamatergic plasticity that may further enhance the abuse-related effects of cocaine (Fitzgerald et al., 1996; Kauer and Malenka, 2007; Stelly et al., 2016). In a recent study by Stelly et al. (2016), rats first underwent a resident-intruder social defeat paradigm in conjunction with corticosterone injections, and then cocaine rewarding effects were assessed using a conditioned place preference (CPP) procedure. Repeated social defeat selectively enhanced long-term potentiation (LTP) of N-Methyl-D-aspartic acid receptors (NMDARs) in the VTA. This LTP manifested as enhanced VTA dopaminergic neuron firing in response to cocaine-associated cues during CPP only in the stressed group. This additional dopaminergic burst was interpreted as enhancing the conditioned stimulus-response relationship between drug-associated cues and the abused drug that may be involved in drug relapse (Stelly et al., 2016). These results suggest stress-induced glutamatergic plasticity of NMDAR and subsequent enhancement of cocaine abuse-related effects may be attenuated in the VTA by a GC antagonist. Deletion of *nuclear receptor subfamily 3, group C, member 1 (nr3c1)*, a gene encoding a GR, blunted cocaine reinforcement in a drug self-administration procedure and VTA dopaminergic firing (Ambroggi et al., 2009; Barik et al., 2013). These results provide further evidence that GRs modulate VTA dopaminergic plasticity that directly impacts the abuse-related effects of cocaine.

**Figure 2:**
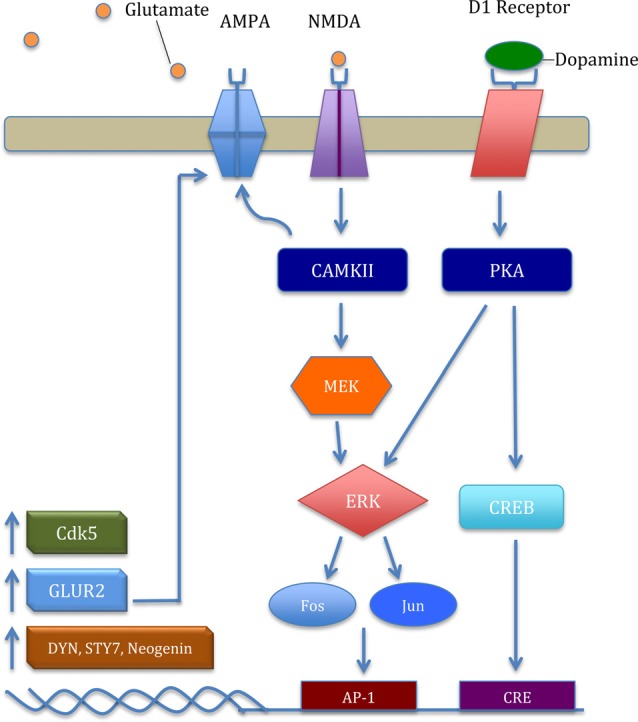
The general mechanism of the extracellular signal-regulated kinase (ERK) pathway and key downstream products important in cocaine addiction. Glutamate binding excites the N-Methyl-D-aspartic acid receptor (NMDAR) and upregulates intracellular calcium. Excitation provokes a signaling cascade, upregulating transcription factors Fos and Jun. Subsequently, increased ΔFosB acts on activator protein-1 (AP-1) and upregulates transcription and translation of cyclin-dependent kinase 5 (CDK5), GLUR2, dynorphin (Dyn), synaptotagmin VII (Syt7), and neogenin. CDK5 mediates localization and GLUR2-mediated plasticity in α-amino-3-hydroxy-5-methyl-4-isoxazolepropionic acid receptor (AMPAR) through phosphorylation of δ-Catenin (Poore et al., 2010). Increased GLUR2 upregulates the ERK pathway in a positive-feedback type manner through increased AMPAR in the nucleus accumbens (NAcs). Moreover, increased D1 receptor activation upregulates protein kinase A (PKA), phosphorylating the transcription factor cAMP response element binding protein (CREB), leading to further increase in CDK5 and GLUR2 protein levels.

Accumulating evidence suggests one molecular mechanism by which both stress and drugs of abuse impact glutamatergic plasticity in the mesolimbic pathway is through extracellular signal-regulated kinases (ERK). For example, stress exposure increased inositol 1,4,5-trisphosphate receptors (IP3R) sensitization that was mediated by protein kinase A (PKA), an upstream activator in ERK pathway (Vanhoutte et al., 1999; Stelly et al., 2016; Figure 2). Consistent with these previous results, social-defeat stress increased ERK signaling in the VTA (Yap et al., 2015). Moreover, ERK signaling appears to rely on the relative ratio of α-amino-3-hydroxy-5-methyl-4-isoxazolepropionic acid receptors (AMPARs) and NMDARs. For example, stress exposure increases the AMPA/NMDA ratio in the VTA (Saal et al., 2003; Dong et al., 2004). However, inhibition of ERK activation has produced equivocal results on the abuse-related effects of cocaine. Administration of SL327, a mitogen-activated protein kinase (MEK) inhibitor used to inhibit ERK, decreased both context and cocaine-induced CPP (Valjent et al., 2000, 2006; Pan et al., 2011). This trend may be indicative of neuroadaptive changes post ERK inhibition. In contrast, administration of U0126, another MEK inhibitor, directly into the VTA enhanced both context and cocaine cue-induced reinstatement in non-stressed rats (Lu et al., 2004, 2009). However, in rats undergoing social stress first, U0126 directly into the VTA attenuated stress-enhanced cocaine locomotor sensitization (Stelly et al., 2016). Taken together, the role of ERK activation in cocaine’s abuse-related effects seems fundamental to understanding downstream physiological and behavioral alterations initiated in the VTA.

### CRF-R1 Modulation

Corticotropin-releasing factor acting at type 1 receptor (CRF-R1) has also emerged as one potential molecular mechanism linking stress and drug abuse. For example, intermittent social defeat stress elicits CRF release in the VTA (Holly et al., 2016). Furthermore, social defeat stress or intra-VTA CRF enhanced the abuse-related effects of cocaine in rats (Boyson et al., 2014; Leonard et al., 2017). Consistent with these previous findings, administration of a CRF antagonist before each social defeat stress attenuated both cocaine-induced locomotor sensitization and escalated cocaine self-administration in rats (Boyson et al., 2011). However, CRF antagonists also decrease escalated cocaine self-administration in non-stressed rats (Specio et al., 2008) suggesting the role of CRF on interactions between social stress and cocaine abuse-related effects have not been fully elucidated. Further complicating the role of CRF in cocaine reinforcement are results from nonhuman primates demonstrating a CRF antagonist does not attenuate cocaine self-administration (Mello et al., 2006). In congruence with this observation, the CRF antagonist verucerfont failed to attenuate alcohol craving in anxious alcoholic women, despite blocking HPA axis responsivity to dexamethasone (Schwandt et al., 2016). Overall, in contrast to the preclinical reports using rodents, nonhuman primate and clinical results do not provide compelling evidence for a significant role of CRF in altering the abuse-related effects of abused drugs in either stress or non-stressed research subjects.

## Nucleus Accumbens NAcs

### Increased LTP From D1 Activation

In addition to drug-induced changes in the VTA, chronic cocaine use and stress exposure can directly alter the NAcs (Wolf and Ferrario, 2010; Koya and Hope, 2011). Preclinical models show cocaine-induced morphological changes in dendritic spine density and greater AMPAR/NMDAR firing in the NAcs after administration alone (Wolf and Ferrario, 2010; Koya and Hope, 2011). Furthermore, chronic stress may alter relapse and self-administration *via* epigenetic modifications to histone dimethyltransferase G9a in the NAcs (Anderson et al., 2018). In addition to drug and stress induced changes in the NAcs, chronic stress exposure may further substance abuse *via* a feedback loop with the VTA. The D1 receptor is a G_s_-protein coupled post-synaptic receptor that is linked to upregulation of FBJ murine osteosarcoma viral oncogene homolog B (ΔFosB), cAMP response element binding protein (CREB), and cyclin-dependent kinase 5 (CDK5; Catalano et al., 2009; Lebel et al., 2009; Zhang et al., 2002). Increased D1 receptor activation leads to upregulated glutamatergic receptors in the NAcs (Chao et al., 2002; Mangiavacchi and Wolf, 2004). In addition, increased D1 activation attenuates GABA-B, a metabotropic transmembrane receptor, inhibition due to changes in adenosine levels after cocaine exposure in the VTA (Bonci and Williams, 1996). Reduced inhibition by GABA-B can subsequently increase LTP (Nicola et al., 2000) and decrease long-term depression (LDP) leading to increased synaptic plasticity in the NAcs (Bonci and Williams, 1996; Nicola et al., 2000; Fourgeaud et al., 2004). NAcs inhibitory neurons can project back to the VTA, resulting in a possible feedback loop of increased neurogenic excitability and DA release (Omelchenko and Sesack, 2009; Xia et al., 2011). The increase in potentiation further excites DA cells, causing DA release (Gonon and Sundstrom, 1996; Gonon, 1997). This theory aligns with recent data suggesting increased DA release after CGP55845 administration, a GABA-B antagonist (Melchior et al., 2015). Subsequently, greater DA in the synapse reduces D1 DA receptor availability in the ventral striatum according to recent PET scans (Martinez et al., 2009). Additional research is needed to support a pattern of a positive feedback loop and greater VTA response to the drug. Furthermore, stress-induced cocaine seeking is initiated by GABA-B receptor-dependent CRF actions in the VTA (Blacktop et al., 2016). Although this modulation by stress is carried out in the VTA, effects of GABA-B and CRF interactions are exerted in the postsynaptic membrane in the NAcs. Additional evidence suggests brain-derived neurotrophic factor (BDNF) may mediate neuronal excitability through activation of tropomyosin receptor kinase B (TrkB) receptors in the NAcs (Berton et al., 2006). Lobo et al. (2010) found a loss of TrkB receptors, mimicked through upregulation of D2 neurons, lead to decreased cocaine reward; in contrast, upregulation of D1 excitability showed an increase in cocaine reward. In addition to BDNF’s mediating role, stress is implicated in facilitation of further synaptic adaptations in the NAcs. To this end, Chaudhury et al. (2013) demonstrated that repeated social defeat stress may induce VTA DA neuron phasic firing to the NAcs in mice. These data suggest that stress-induced phasic firing of the VTA may augment synaptic excitability in the NAcs of cocaine-addicted brains (Chaudhury et al., 2013).

**Figure 3:**
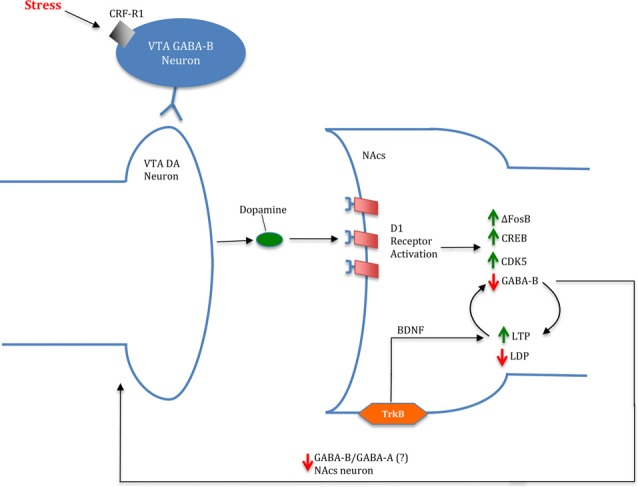
Image of a potential mechanism for stress- and cocaine-induced drug dependence *via* a feed-forward cycle in the NAcs. In the presence of stress, ventral tegmental area (VTA) DA release is upregulated resulting in increased D1 receptor activation. Cortisol is implicated in increasing DA release through corticotropin-releasing factor acting at type 1 receptor (CRF-R1) binding to gamma-aminobutyric acid (GABA)-B VTA neurons acting on VTA DA neurons. Increased DA levels promote D1 activation leading to an increase in ΔFosB, CREB and CDK5 levels in the NAcs. Moreover, D1 activation is linked to decreased GABA-B activation in the NAcs, resulting in greater long-term potentiation (LTP): long-term depression (LDP). Attenuation of GABA projections from NAcs to the VTA is suggested to further DA release; however, the particular projection (GABA-A/GABA-B) is currently unknown. Furthermore, brain-derived neurotrophic factor (BDNF) is implicated in contributing to LTP in the NAcs through activation of tropomyosin receptor kinase B (TrkB) receptors.

The proposition that stress exerts effects through inhibition of positive feedback is not fully supported in the extant literature. For example, Sinha (2008), reported that chronic stress inhibits DA synthesis in the NAcs. However, it is well supported that GC concentrations directly correlate with extracellular DA release (Brake et al., 2004; Sinha, 2008). Although DA synthesis may be inhibited by chronic stress, cocaine sensitization has been repeatedly shown to increase by gene and protein regulators such as ΔFosB, CREB and CDK5 (Kelz et al., 1999; Bibb et al., 2001; McClung and Nestler, 2003; Mattson et al., 2005). Therefore, the combined data leads us to conclude that stress increases drug addiction susceptibility through increased sensitization in a positive feedback manner (Figure 3). Furthermore, the literature suggests that stress perpetuates drug dependence through allostasis by reinforcement in an analogous feedback manner (Koob and Le Moal, 2001; Ahmed et al., 2002). Taken together, the available findings collectively suggest that stress may mediate drug dependence at multiple levels, through positive feedback mechanisms.

## Conclusion and Future Directions

Although this mini-review article has focused on the effects of stress on the mesolimbic DA pathway, the effects of stress on other brain regions implicated in substance use disorders are important considerations beyond the capacity of this brief synopsis. For example, GRs are highly expressed in the PFC. GCs can act locally in the PFC to modulate cognitive impairments in working memory due to acute stress (Butts et al., 2011). Similar to GC effects on the mesolimbic DA pathway, corticosterone administered directly into the PFC can increase DA efflux (Butts et al., 2011). However, despite the relevant function of the PFC in substance use disorders (Volkow et al., 2016), relatively little research has been done to determine the extent to which molecular intermediaries such as ΔFosB, CREB, or CDK5 are involved in the PFC with regard to stress-induced enhancement of cocaine abuse-related effects.

Collectively, this mini-review article details three potential molecular mechanisms relating DA and GC interactions as they relate to stress-induced enhancement of cocaine abuse-related behaviors. In all three mechanisms, stress-induced GC release and subsequent activation of GRs primes the mesolimbic DA pathway. The overall net effect is enhanced abuse-related effects of cocaine and enhanced susceptibility of progressing to a cocaine use disorder diagnosis (Sinha, 2008). Thus, stress may serve as a positive feedback mechanism in the NAcs for enhancing the susceptibility to, or progression to, substance use disorder.

## Author Contributions

DM and GN discussed ideas for the initial submission. DM led the literature search and discussed results with GN. GN advised on direction and additional resources. DM wrote the majority of the initial manuscript and GN provided revisions and reformatting of content. MB provided substantial editorial comments following the first stage of review including writing additional content and recommendations on revisions for existing content. All individuals have approved the final version and agree to be responsible for the content.

## Conflict of Interest Statement

The authors declare that the research was conducted in the absence of any commercial or financial relationships that could be construed as a potential conflict of interest.
